# Molecular typing of *Coxiella burnetii* from animal and environmental matrices during Q fever epidemics in the Netherlands

**DOI:** 10.1186/1746-6148-8-165

**Published:** 2012-09-18

**Authors:** Arnout de Bruin, Pleunie TW van Alphen, Rozemarijn QJ van der Plaats, Lianne ND de Heer, Chantal BEM Reusken, Bart J van Rotterdam, Ingmar Janse

**Affiliations:** 1 National Institute for Public Health and the Environment (RIVM), Centre for infectious Disease Control (Cib), Laboratory for Zoonoses and Environmental Microbiology (LZO), PO Box 1, Bilthoven, 3720, BA, the Netherlands; 2 Laboratories for Pathology and Medical Microbiology (PAMM Foundation), PO Box 2, Veldhoven, 5500, AA, the Netherlands

**Keywords:** *Coxiella burnetii*, Q fever, Molecular typing, MLVA, Environment, Goat, Sheep

## Abstract

**Background:**

The bacterium *Coxiella burnetii* has caused unprecedented outbreaks of Q fever in the Netherlands between 2007 and 2010. Since 2007, over 4000 human cases have been reported, with 2354 cases in 2009 alone. Dairy goat farms were identified as most probable sources for emerging clusters of human Q fever cases in their vicinity. However, identifying individual farms as primary source for specific clusters of human cases remains a challenge, partly due to limited knowledge of the different *C. burnetii* strains circulating in livestock, the environment and humans.

**Results:**

We used a multiplex multi-locus variable number of tandem repeats analysis (MLVA) assay to investigate the genotypic diversity of *C. burnetii* in different types of samples that were collected nationwide during the Dutch Q fever outbreaks between 2007 and 2010. Typing was performed on *C. burnetii* positive samples obtained from several independent studies investigating *C. burnetii* presence in animals and the environment. Six different genotypes were identified on 45 farm locations, based on sequence-confirmed estimates of repeat numbers of six MLVA markers. MLVA genotype A was observed on 38 of the 45 selected farm locations in animals and in environmental samples.

**Conclusions:**

Sequence confirmation of the numbers of tandem repeats within each locus and consensus about repeat identification is essential for accurate MLVA typing of *C. burnetii.* MLVA genotype A is the most common genotype in animal samples obtained from goat, sheep, and rats, as well as in environmental samples such as (aerosolized) dust, which is considered to be the major transmission route from animals via the environment to humans. The finding of a single dominant MLVA genotype in patients, the environment, and livestock complicates accurate source-finding. Pinpointing individual sources in the Netherlands requires discrimination of genotypes at a higher resolution than attained by using MLVA, as it is likely that the dominant *C. burnetii* MLVA type will be detected on several farms and in different patients in a particular area of interest.

## Background

Q fever, caused by the bacterium *Coxiella burnetii*, has been a public health problem in the Netherlands between 2007 and 2010 [1-4]. Between 2005 and 2007, before the first documented Q fever outbreak in the Netherlands, *C. burnetii* related abortions were reported on a number of commercial dairy goat farms in a rural area in the southeast of the Netherlands [1]. In 2007, a Q fever outbreak was reported in humans in the same rural area, which expanded to other areas in the Netherlands in subsequent years [3,4]. The 2007 outbreak initiated source-finding investigations in the affected areas during the subsequent epidemics in 2008 and 2009. The investigations were initiated by several Municipal Health Services, in close collaboration with the Netherlands Food and Consumer Product Safety Authority (NVWA) and the National Institute for Public Health and the Environment (RIVM). Vaginal swabs from goats and sheep and surface swabs from stables, that were obtained during these source-finding studies, revealed that *C. burnetii* DNA is present on most dairy farms suspected of being a source for human Q fever cases in their vicinity [5]. In addition, epidemiological studies indicated commercial dairy goat farms as most likely sources for human Q fever infection in the Netherlands [1,5,6]. Genotyping of *C. burnetii* DNA obtained from human, animal and environmental samples could yield further insight in the possible link between the (clusters of) human Q fever cases and *C. burnetii* positive farms. To enable such studies, genotypic characterization of *C. burnetii* strains circulating in the different reservoirs is needed.

A number of different molecular typing methods have been developed to analyze genetic variability among *C. burnetii* laboratory isolates. One of the first molecular typing methods for *C. burnetii* is based on restriction fragment length polymorphism (RFLP) in combination with pulse field gel electrophoresis (PFGE) [7,8]. More recently, PCR based methods were developed for molecular typing of *C. burnetii* strains, including multi-locus variable number of tandem repeats analysis (MLVA) [9,10], multispacer sequence typing (MST) [11], and single nucleotide polymorphism (SNP) [12,13]. Previous studies of *C. burnetii* genotypes during the epidemics in the Netherlands showed low genetic diversity, and domination by one genotype in human and animal samples [14,15]. In this study, we expand these analyses by investigating environmental samples, which are thought to play an important role in transmission of *C. burnetii* from animals to humans. We investigated farms that have been identified as potential sources for human Q fever in the Netherlands. Moreover, we included regions in the Netherlands that had not been studied previously.

## Results

The selection of samples for molecular typing using MLVA was based on qPCR assays that have been used for the detection of *C. burnetii* in several studies [5,16,17]. DNA extracts showing Cq values of 31 or lower for *C. burnetii* target *IS1111* in qPCR assays, were selected for molecular typing using a newly developed multiplex MLVA assay. Attempts to amplify DNA from samples with higher Cq values (lower *C. burnetii* DNA content) were unsuccessful. Characteristics of the two developed multiplex MLVA PCR assays are described in Table 1.

**Table 1:** Characteristics of the two multiplex MLVA assays

**Locus**	**Primers**	**MLVA primer sequence and label (5′-3′)**	** Repeat sequence**	**Repeat length (bp)**	** Sequencing primers (5′-3′)**
	*Multiplex assay 1*				
Ms27 (Cox2)	Forward primer	**NED**-CGCTATTTTTTCAGTTTTGAGTAA	TGAAGA	6	CAGTGCGTCCAGATTTCATTG
	Reverse primer	GTCGCAAACGTCGCACT			GTCCGGATACAGCTTGAAAAGT
Ms28 (Cox5)	Forward primer	**VIC**-CGATGACGACAAAAAAGACT	TAAGAA	6	GCAGAAAAAGAACATGAATGTGATTGTG
	Reverse primer	GCGGTAATTACTGTAAATAAATACAAAGACA			GCCGGTAMCCTTCTCTAAATATTGCAA
Ms34 (Cox1)	Forward primer	**6FAM**-ATCAGCGACTCGAAGAAAAA	GAAAAG	6	CCAGTATCTCGTACGTCTCRATTT
	Reverse primer	AGGGTGACTTTTTCACTTAAAG			CGTTTGAACACGCAACTGTTTT
	*Multiplex assay 2*				
Ms20b	Forward primer	**VIC**-TGTAACAGCACCGCCTGA	GGAAGAAGCGCCACCCG	33	ACAGGTGAGTCGCCATTAACG
	Reverse primer	GCTTTGCCCTTTCCTTGATTTTC	AAATAGGGTTGCCTCC		CCATTGGGATCAAGTTCATGACTAT
Ms24 (Cox4)	Forward primer	**NED**-CCATTGTGTAATTGACATGAAGAA	GACGGAA	7	TATGCGCATCTTCTCGGAGCA
	Reverse primer	GCCACACAACTCTGTTTTCAG			GCGCTCCTTCCTCCTGTAAG
Ms31 (Cox7)	Forward primer	**6FAM**-CCGGTATTCTAACCAACTGAAC	CAGAGGA	7	AGATAAAAAGAAAAAGCAACCCGTGAA
	Reverse primer	GAATCCCTCAGCACCCATTC			GGGTGCGTTTCCAAAAATAGTATAGG

Per assay, three different primer sets, labeled with different fluorescent dyes, target three different loci. Loci are indicated by their previously published names: Ms [9], and Cox [10]. The sequencing primer sets were used for sequencing MLVA motifs.

The numbers of tandem repeats calculated from PCR fragment sizes obtained for each locus were confirmed by sequencing (Table 2). Significant discrepancies between sequencing results and PCR fragment sizes were found for markers Ms31 and Ms34. For these markers, the number of repeats that was calculated based on PCR fragment sizes was approximately one repeat lower than the number obtained from sequencing. In addition, for markers Ms24 and Ms31 one of the repeats that was included in the repeat count was not located immediately adjacent to the other repeats. Repeat numbers thus calculated are in accordance with the number of tandem repeats identified for the *C. burnetii* Nine Mile RSA 493 phase I strain in other studies [14,15]. Therefore, sequencing results guided correct estimations of the numbers of tandem repeats in the MLVA genotyping of our samples.

**Table 2:** Results of fragment analyses and sequencing of PCR products obtained from samples of commercial dairy farms

**Locus**	**Fragment**	**PCR product length (bp)**		**Number of tandem repeats**	
		**Fragment analysis**	**Sequencing**	**Fragment analysis**^**1**^	**Sequencing**
Ms27	1	285	282	2.4	2.0
	2	290	288	3.3	3.0
	3	295	294	4.2	4.0
Ms28	1	189	190	2.8	3.0
	2	201	202	4.8	5.0
	3	207	208	5.8	6.0
	4	213	214	6.8	7.0
Ms34	1	107	112	1.2	2.0
	2	113	118	2.2	3.0
	3	124	130	4.1	5.0
	4	136	142	6.1	7.0
	5	143	148	7.1	8.0
	6	148	153	8.1	9.0
Ms20b	1	252	258	4.4	4.5
	2	269	273	4.9	5.0
	3	349	354	7.3	7.5
Ms24	1	165	163	9.3	9.0
	2	179	177	11.3	11.0
	3	192	191	13.1	13.0
	4	287	289	26.7	27.0
Ms31	1	130	136	2.1	3.0
	2	145	150	4.3	5.0

^1^The numbers of tandem repeats from fragment sizes were calculated by dividing the sizes of the measured PCR products minus primer binding sites and flanking regions by the corresponding repeat length.

A total of 190 samples (94 environmental and 96 animal samples), originating from 45 farm locations were successfully genotyped. Based on six markers, a total of six different MLVA genotypes (A-F) could be discriminated (Table 3). Overall, the most common *C. burnetii* type observed in all samples was MLVA genotype A, followed by genotype B, genotype E, genotype D, genotype F, and genotype C. The number of MLVA genotypes per animal or environmental matrix and farm type is presented in Table 4. The most abundant genotype A was found in all animal and environmental matrices. Genotype B was observed in vaginal swabs and surface swabs, while genotypes C and D were observed in vaginal swabs from goats only. Genotype E was found in spleens from rats, but not in any of the animal samples obtained from goats or sheep. Genotype F was observed in surface swabs of a single dairy sheep farm only.

**Table 3:** ***Coxiella burnetii *MLVA types observed in 96 animal and 94 environmental samples obtained from 45 dairy farm locations in the Netherlands**

***C. burnetii *MLVA genotype**	**Origin**	**Number of samples**			**Number of repeats**					
		**Total**	**Animal**	**Environment**	**MLVA multiplex assay 1**			**MLVA multiplex assay 2**		
					**Ms27**	**Ms28**	**Ms34**	**Ms20b**	**Ms24**	**Ms31**
A	Samples	145 (5)	69	76 (5)	3	3	7	7.5	11	3
B	Samples	24	11	13	3	3	8	7.5	11	3
C	Samples	2	2	0	2	7	8	5	13	3
D	Samples	9	9	0	4	5	2	4.5	9	3
E	Samples	10 (5)	5	5 (5)	2	7	9	5	13	3
F	Samples	5	0	5	2	3	3	7.5	11	3
Nine Mile RSA 493 phase I	Cultivation	1	1	0	4	6	5	5	27	5
Nine Mile RSA 493 phase I	*in silico*	n.a.	n.a.	n.a.	4	6	5	5	27	5
Dugway 5 J108-111	*in silico*	n.a.	n.a.	n.a.	4	4	3	7	4	3
RSA 331	*in silico*	n.a.	n.a.	n.a.	3	3	3	7,5	6	2
CbuG_Q212	*in silico*	n.a.	n.a.	n.a.	3	4	2	5	7	4
CbuK_Q154	*in silico*	n.a.	n.a.	n.a.	4	5	2	4.5	8	3

n.a. = not applicable.

Numbers between brackets indicate samples with both genotype A and E. The calculation of the numbers of repeat units per marker are guided by sequencing results.

**Table 4:** ***Coxiella burnetii *MLVA types observed in three animal and four environmental matrices obtained from 45 farm locations in the Netherlands**

**Matrix type**	** Sample type**	** Origin**	**Sample size**	**MLVA genotypes**						
				**A**	**B**	**C**	**D**	**E**	**F**	**A + E**^**a**^
Animal	Vaginal swabs	Goats	63 (15)	46 (11)	6 (2)	2 (1)	9 (1)			
		Sheep	19 (6)	14 (5)	5 (1)					
	Placentas	Goats	2 (2)	2 (2)						
		Sheep	1	1						
	Spleens	Rats	11 (4)	6 (3)				5 (1)		
Environment	Surface swabs	Goat farms	69 (17)	53 (13)	13 (3)					3 (1)
		Sheep farms	9 (3)	4 (2)					5 (1)	
	Manure	Goat farms	1	1						
	Milk unit filters	Goat farms	13 (7)	11 (6)						2 (1)
	Aerosols	Goat farms	2 (2)	2 (2)						

^a^both genotype A and E present in sample.

Samples were obtained from 34 dairy goat farms, 9 (non-dairy) sheep, one cattle farm, and one petting zoo. The number of farms on which the samples were collected is displayed between brackets.

**Figure 1:**
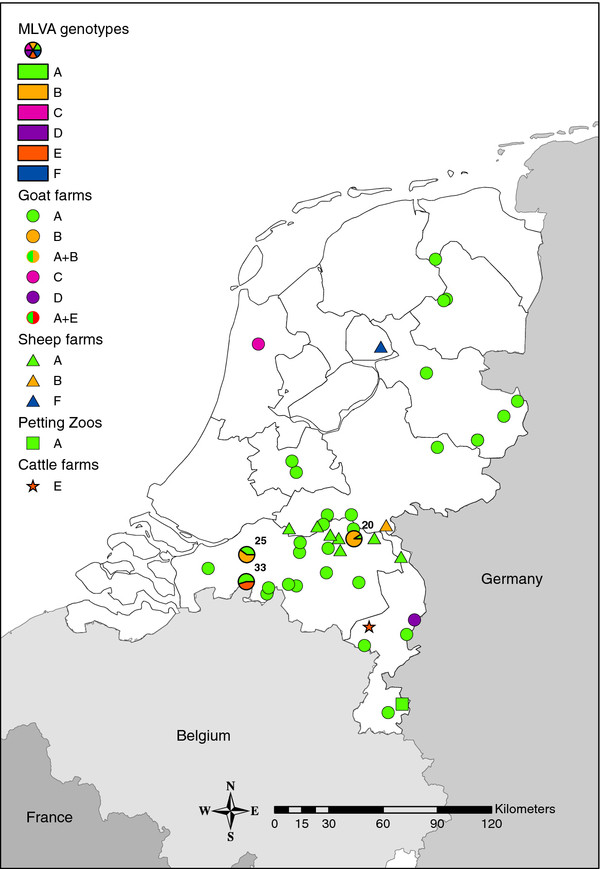
**MLVA genotypes, indicated by coloured symbols, on 45 dairy farm location in the Netherlands.** Dairy goat farms are indicated by circles, (non-dairy) sheep farms by triangles, petting zoos by squares, and cattle farms by stars. The proportion of MLVA genotypes on farms 20, 25 and 33, where more than one genotype was encountered, are indicated by pie charts.

On most farms, only one single *C. burnetii* MLVA genotype was detected. Genotype A was encountered as a single genotype on most farms (36 locations), genotype B was the single genotype on two farms, and genotypes C, D, E and F were single genotypes on four farms (Figure 1). On three farms, additional MLVA genotypes were encountered besides the most common genotype A. On farm 20, MLVA genotypes A and B were observed in separate vaginal swab samples, while type B was the only genotype in surface swabs. On farm 25, MLVA type A was observed in milk filters, while MLVA type B was observed in surface swabs. On farm 33, both MLVA genotypes A and E were observed as mixed genotypes in milk filters and surface swabs.

## Discussion

The Q fever outbreak of 2007 was the first documented outbreak in the Netherlands. A case control study in 2007 revealed several risk factors for acquiring Q fever, however, a direct link with a particular source could not be established [3]. In this study we show that the most common MLVA genotype in both animal and environmental samples obtained from 45 farm locations is MLVA genotype A. The environmental sampling categories, surface swabs and aerosols, are of particular interest for establishing a source hypothesis of *C. burnetii* infection, since they may provide the link between *C. burnetii* in animals and humans. *Coxiella burnetii* laden dust can originate from the decomposition of *C. burnetii* contaminated aerosols and vice versa, re-aerosolisation of contaminated dust might occur. Contaminated aerosols are regarded as one of the most important transmission routes for *C. burnetii* to humans, especially when environmental conditions for aerosol dispersion are favourable [3,16,18,19]. Surface swabs were collected on 20 out of the 45 sampling locations and showed the occurrence of MLVA genotype A at 16 of these locations. Two aerosol samples collected at two of these locations were suitable for typing and MLVA genotype A was observed in these samples as well. As MLVA genotype A was also the dominant genotype in animal samples obtained from goats, sheep, and rats (Table 4), these data show that the most common genotype can be detected in both the animal hosts and the environmental matrices that are considered to play a dominant role in the direct or indirect transmission from animals to humans.

The finding of a single most common *C. burnetii* MLVA genotype in both environmental and animal samples in this study, supports the findings of two other MLVA genotyping studies in the Netherlands [14,15]. The 33 clinical samples that were successfully typed by Tilburg *et al.*[15] using a panel of six MLVA markers, revealed a most common MLVA type referred to as MLVA genotype G (in 18 out of 33 samples). The four MLVA markers (Ms24, Ms27, Ms28, and Ms34) that can be compared to our study showed tandem repeats numbers that were identical to our most common MLVA genotype A. The most common MLVA genotype in the veterinary study by Roest *et al.*[14] was referred to as MLVA genotype CbNL01, and was detected in 112 out of 126 animal samples obtained from 14 dairy goat farms, one dairy cattle farm and two sheep farms. Roest *et al*. [14] used 11 MLVA markers for genotyping *C. burnetii,* as described by [9]. Five of these markers, Ms24, Ms27, Ms28, Ms31, and Ms34, can be compared to our study, and MLVA genotype CbNL01 showed identical numbers of tandem repeats within these loci when compared to our most common MLVA genotype A [14].

The MLVA loci that can be compared between the three molecular typing studies of *C. burnetii* during the Q fever epidemic in the Netherlands show identical results for the numbers of tandem repeats within these loci for the most common genotypes. However, even when the different MLVA assays investigate the same markers, differences in primers used for amplification and other laboratory-specific conditions may affect the outcome of the analyses. In addition, the scoring of the numbers of tandem repeats may differ between individual researchers. This was illustrated by the outcome of an interlaboratory comparison with 7 European participants [20]. Finally, the sequence information regarding the repeat motif for a number of MLVA loci was not published [9]. This lead to the development of new primers for marker Ms20 in our study, which target a slightly different region than published by [9,21]. Therefore, comparisons of *C. burnetii* MLVA typing between laboratories requires a consensus approach, and confirmation of the number of tandem repeats by sequencing will play an essential role in providing the basis for confident calculations. This is of particular importance when the differences between MLVA genotypes, both within and between studies, are rather small.

Establishing a direct relationship between (clusters of) human Q fever cases and a single (or a cluster) of *C. burnetii* affected farms remains a challenge. Two epidemiological studies performed during the Q fever outbreaks in 2008 and 2009 in the Netherlands showed a strong association between clusters of human Q fever cases and a commercial dairy goat farm [6] and a non-dairy sheep farm [22]. Although these studies focused on the best described clusters of human Q fever cases observed during the outbreaks, information about the number of *C. burnetii* genotypes circulating in patients, the environment, and potential veterinary sources in the Netherlands was not available at that time. Molecular typing of all samples obtained from animals and surface swabs from the dairy goat farm that was identified as the most probable source of a cluster of human Q fever cases [6], revealed the presence of MLVA genotype A only. Unfortunately, molecular typing of the samples obtained from the non-dairy sheep farm that had been identified as the primary source for the cluster of human Q fever cases in its vicinity in 2009 [22], was not successful due to low *C. burnetii* DNA content (Cq values for target *IS1111* >31). Investigations of *C. burnetii* MLVA genotypes in patients [15], dairy live-stock [14] mentioned earlier, and environmental and animal matrices in this study, showed that a single *C. burnetii* MLVA genotype is dominant in the Netherlands. Based on these data we conclude that the resolution attained by using MLVA is insufficient for pinpointing individual sources in most cases, as it is likely that the dominant *C. burnetii* type will be detected on several farms and in different patients in a particular area.

Another factor that complicates source-finding investigations, is that these studies are often biased by the selection of farms. For instance, in 2009 about 350,000 goats were registered on 3916 farms in the Netherlands. About 274,000 goats were distributed over 370 large commercial dairy goat farms.

The number of sheep was even larger in 2009, with over one million sheep present in the Netherlands, distributed over 12,833 registered farms (Statistics Netherlands: CBS). Only a small subset of these farms were selected for source-finding investigations in 2008 (29 farms) and 2009 (56 farms). Therefore, the number of MLVA genotypes observed in this study is an underestimation, because only 9.2% of commercial dairy goat farms and less than 1% of sheep farms present in the Netherlands in 2009 were included. Since dairy goat farms were quickly identified as the most probable sources for human Q fever in the Netherlands [1,5,6,23], the numbers of investigated dairy goat farms were much higher when compared to other potential sources for human Q fever (e.g. sheep or cattle). Primarily, those farms located in close proximity to human Q fever cases were selected. Within these clusters, farms were encountered with large numbers of samples positive for *C. burnetii*, as well as farms without any positive samples [5]. Furthermore, it is an interesting observation that rats, a potential reservoir for *C. burnetii*[17], collected on a cattle farm showed a different MLVA genotype (type E) in comparison to rats collected on a dairy goat farm (MLVA genotype A). Genotype E was also encountered in environmental samples obtained from a dairy goat farm, but only when genotype A was also detected. MLVA genotype F was observed on a single dairy sheep farm only and not on dairy goat farms, or non-dairy sheep farms. Although these data may suggest a preferential association of particular genotypes with specific hosts, further studies are needed to substantiate such a link. Finally, adequate source-finding studies for accurate source-attribution in the Dutch situation require a more detailed analysis of the strains present in animals, the environment and humans. Such studies would benefit from a method that enables strain differentiation at a higher resolution, both in time and in space. This requires improved genotyping methods specifically designed for the genotypes encountered in the Dutch outbreaks.

## Conclusions

MLVA genotype A is the most common genotype in animal samples obtained from goat, sheep, and rats, and in environmental samples that are considered to be a major transmission route from animals to humans. The finding of a single most common genotype in patients, environment, and livestock complicates accurate source finding. The resolution attained by using MLVA is insufficient for pinpointing individual sources, as it is likely that the dominant *C. burnetii* type will be detected on several farms and in different patients in a particular area. Confident MLVA-typing of *C. burnetii* will be improved by the confirmation of the number of tandem repeats by sequencing for confident calculations of the number of tandem repeats. Moreover, it is very important that a consensus, about counting, scoring and identifying the number of tandem repeats for each locus, is reached for strengthening the outcome of MLVA analyses.

## Methods

### Development of a multiplex MLVA assay for C. burnetii using published loci

Several MLVA genotyping assays have been described for *C. burnetii*[9,10,24]. The MLVA assay described in this study includes repeat regions that are used by other authors as well. The use of a multiplex MLVA assay as described by [24] has the advantages of reduced sample usage and analysis efforts. Due to difficulties in repeating the published multiplex protocol, and to enable the analysis of additional markers, we designed novel primers for MLVA amplification. Primer design was based on the genome sequences from *C. burnetii* Nine Mile RSA 493 phase I strain (Genbank accession number AE016828), *C. burnetii* strains Dugway (Genbank accession number CP000733), RSA331 (CP000890), CbuG Q212 (CP001019), and CbuK Q154 (CP001020). For most regions, the repeats were identical to those in other MLVA protocols. However, for Ms20 we used a different repeat compared to the one presented by [9]. This repeat was identified by using Tandem Repeat Finder software [25] and should be named Cbu1941_ms20b_33bp_5U_273bp, according to the format proposed by [9]. Since the repeat motif and amplified region differ, a direct comparison of repeats between these studies is not possible.

Using the software package Visual Oligonucleotide Modeling Platform version 7.6.19 (DNA software Inc., Ann Arbor, MI), primers were developed for two multiplex reactions, amplifying a total of six markers (Table 1). Primers were obtained from Biolegio (Nijmegen, the Netherlands). Each multiplex amplification reaction was carried out in 20 μl, containing 0.2 μM primers, 4 μl of template DNA, and 16 μl of Qiagen multiplex PCR mix (Venlo, the Netherlands). MLVA multiplex PCR assays were carried out on a PCR-express machine (Thermo-Scientific, Breda, the Netherlands) using the following conditions: 15 min. at 95°C (DNA polymerase activation), followed by 40 cycles of 30 s of denaturation at 95°C, 90 s of annealing at 55°C, and 1 min. extension at 72°C. Finally, a ten min. incubation step was performed at 72°C.

PCR products were purified by adding 2 μl of ExoSAP-IT (Affymetrix, Germany) to 5 μl of PCR product, followed by incubation at 37°C for 15 min., and inactivation at 80°C for 15 min. Separation of PCR fragments (2 μl PCR product + 10 μl of LIZ 500 Genescan size standard) was performed on an ABI 3700 DNA sequencer (Applied Biosystems, Foster City, CA) using the standard GeneScan module. GeneScan data were imported into Genemarker (SoftGenetics, USA) for analysis.

### Calculation of the number of tandem repeats for each marker

PCR product lengths for each marker were obtained by using Genemarker software. The numbers of tandem repeats were calculated by subtracting primer binding sites and flanking regions from the PCR product size, followed by division by the size of a single tandem repeat. These calculations were based on the published genome sequence, and MLVA and sequencing analysis of the *C. burnetii* RSA 493 Nine Mile strain.

All repeats calculations were confirmed by sequencing of representative samples. Therefore, for each marker, primer pairs were developed to obtain PCR products extending beyond the multiplex MLVA primer binding sites (Table 1). These PCR products were sequenced to calculate the number of repeats within repeat regions. Singleplex reactions for each marker were performed using identical thermocycling reactions as described above. PCR products were sent to BaseClear B.V. (Leiden, the Netherlands) for sequencing both strands. Sequences were imported into BioNumerics (Applied Maths, US) version 6.0 to construct consensus sequences for each marker.

### Selection of samples for molecular typing

DNA extracts selected for MLVA typing originate from animal matrices (spleens from rats and vaginal swabs and placenta material from goat or sheep) and environmental matrices (milk unit filters, manure, surface swabs and aerosols) obtained in stables from dairy goat, (non-dairy) sheep, and cattle farms during several independent Q fever investigations initiated during the outbreaks between 2007 and 2010. These studies included: (i) source-finding investigations initiated by Municipal Health Services in 2008 [5] and 2009, (ii) a study on *C. burnetii* DNA presence in animal and environmental matrices on small ruminant farms in 2009 [16], (iii) a study on *C. burnetii* DNA presence in aerosols collected in stables and in the vicinity of dairy farms in 2009 [De Bruin *et al.,* unpublished results], and (iiii) a study on *C. burnetii* in brown (*Rattus norvegicus*) and black (*R. rattus*) rats, which constitute a potential reservoir for *C. burnetii*[17]. The sampling strategy, sample processing, DNA extraction procedures and qPCR detection in these studies are described elsewhere [5,16,17]. Sampling of animal matrices, such as vaginal swabs from goat, or sheep were carried out by qualified veterinarians of the Netherlands Food and Consumer Product Safety Authority (NVWA). DNA extracts obtained from rats spleens were provided by Dr. Reusken. Procedures and ethical statements regarding animal handling, dissection, and DNA extraction are described in [17].

## Competing interests

The authors declare that they have no competing interests.

## Authors’ contributions

AdB: data analyses, writing and preparation of the manuscript. PTWvA: Sample preparation, DNA extraction, and MLVA analyses of samples RQJvdP: Sample preparation, DNA extraction, qPCR, and MLVA analyses of samples DNdH: Sample preparation, DNA extraction, qPCR, and MLVA analyses of samples CBEMR: co-writer of manuscript, provided DNA of rat spleens for MLVA analyses BJvR: funding and co-writer of the manuscript. IJ: design and set-up of MLVA assays, co-writer of the manuscript. All authors read and approved the final manuscript.

## References

[B1] Roest HI Tilburg JJ van der Hoek W Vellema P van Zijderveld FG Klaassen CH Raoult D The Q fever epidemic in The Netherlands: history, onset, response and reflection Epidemiol Infect 2011 139 1 1 12 10.1017/S0950268810002268 20920383

[B2] van der Hoek W Dijkstra F Schimmer B Schneeberger PM Vellema P Wijkmans C Ter Schegget R Hackert V van Duynhoven Y Q fever in the Netherlands: an update on the epidemiology and control measures Euro Surveill 2010 15 12 20350500

[B3] Karagiannis I Schimmer B Van Lier A Timen A Schneeberger P Van Rotterdam B De Bruin A Wijkmans C Rietveld A Van Duynhoven Y Investigation of a Q fever outbreak in a rural area of The Netherlands Epidemiol Infect 2009 137 9 1283 1294 10.1017/S0950268808001908 19161644

[B4] Enserink M Infectious diseases. Questions abound in Q-fever explosion in the Netherlands Science 2010 327 5963 266 267 10.1126/science.327.5963.266-a 20075230

[B5] de Bruin A de Groot A de Heer L Bok J Wielinga PR Hamans M van Rotterdam BJ Janse I Detection of Coxiella burnetii in Complex Matrices by Using Multiplex Quantitative PCR during a Major Q Fever Outbreak in The Netherlands Appl Environ Microbiol 2011 77 18 6516 6523 10.1128/AEM.05097-11 21784920 PMC3187144

[B6] Schimmer B Ter Schegget R Wegdam M Zuchner L de Bruin A Schneeberger PM Veenstra T Vellema P van der Hoek W The use of a geographic information system to identify a dairy goat farm as the most likely source of an urban Q-fever outbreak BMC Infect Dis 2010 10 69 10.1186/1471-2334-10-69 20230650 PMC2848044

[B7] Heinzen R Stiegler GL Whiting LL Schmitt SA Mallavia LP Frazier ME Use of pulsed field gel electrophoresis to differentiate Coxiella burnetii strains Ann N Y Acad Sci 1990 590 504 513 10.1111/j.1749-6632.1990.tb42260.x 2378472

[B8] Jager C Willems H Thiele D Baljer G Molecular characterization of Coxiella burnetii isolates Epidemiol Infect 1998 120 2 157 164 10.1017/S0950268897008510 9593485 PMC2809385

[B9] Arricau-Bouvery N Hauck Y Bejaoui A Frangoulidis D Bodier CC Souriau A Meyer H Neubauer H Rodolakis A Vergnaud G Molecular characterization of Coxiella burnetii isolates by infrequent restriction site-PCR and MLVA typing BMC Microbiol 2006 6 38 10.1186/1471-2180-6-38 16640773 PMC1488860

[B10] Svraka S Toman R Skultety L Slaba K Homan WL Establishment of a genotyping scheme for Coxiella burnetii FEMS Microbiol Lett 2006 254 2 268 274 10.1111/j.1574-6968.2005.00036.x 16445755

[B11] Glazunova O Roux V Freylikman O Sekeyova Z Fournous G Tyczka J Tokarevich N Kovacava E Marrie TJ Raoult D Coxiella burnetii genotyping Emerg Infect Dis 2005 11 8 1211 1217 16102309 10.3201/eid1108.041354 PMC3320512

[B12] Huijsmans CJ Schellekens JJ Wever PC Toman R Savelkoul PH Janse I Hermans MH Single-nucleotide-polymorphism genotyping of Coxiella burnetii during a Q fever outbreak in The Netherlands Appl Environ Microbiol 2011 77 6 2051 2057 10.1128/AEM.02293-10 21257816 PMC3067327

[B13] Hornstra HM Priestley RA Georgia SM Kachur S Birdsell DN Hilsabeck R Gates LT Samuel JE Heinzen RA Kersh GJ Rapid typing of Coxiella burnetii PLoS One 2011 6 11 e26201 10.1371/journal.pone.0026201 22073151 PMC3206805

[B14] Roest HI Ruuls RC Tilburg JJ Nabuurs-Franssen MH Klaassen CH Vellema P van den Brom R Dercksen D Wouda W Spierenburg MA Molecular epidemiology of Coxiella burnetii from Ruminants in Q fever outbreak, the Netherlands Emerg Infect Dis 2011 17 4 668 675 10.3201/eid1704.101562 21470457 PMC3377418

[B15] Tilburg JJ Rossen JW van Hannen EJ Melchers WJ Hermans MH van de Bovenkamp J Roest HJ de Bruin A Nabuurs-Franssen MH Horrevorts AM Genotypic diversity of Coxiella burnetii in the 2007-2010 Q fever outbreak episodes in The Netherlands J Clin Microbiol 2012 50 3 1076 1078 10.1128/JCM.05497-11 22189106 PMC3295096

[B16] de Bruin A van der Plaats RQJ de Heer DN Paauwe R Schimmer B Vellema P van Rotterdam BJ van Duynhoven Y Detection of Coxiella burnetii on small ruminant farms during a Q fever outbreak in the Netherlands Appl Environ Microbiol 2012 78 6 1652 1657 10.1128/AEM.07323-11 22247143 PMC3298153

[B17] Reusken C van der Plaats R Opsteegh M de Bruin A Swart A Coxiella burnetii (Q fever) in Rattus norvegicus and Rattus rattus at livestock farms and urban locations in the Netherlands; could Rattus spp. represent reservoirs for (re)introduction? Prev Vet Med 2011 101 1-2 124 130 10.1016/j.prevetmed.2011.05.003 21640416

[B18] van der Hoek W Hunink J Vellema P Droogers P Q fever in The Netherlands: the role of local environmental conditions Int J Environ Health Res 2011 21 6 441 451 10.1080/09603123.2011.574270 21563011

[B19] Tissot-Dupont H Amadei MA Nezri M Raoult D Wind in November, Q fever in December Emerg Infect Dis 2004 10 7 1264 1269 10.3201/eid1007.030724 15324547 PMC3323349

[B20] Sidi-Boumedine K Duquesne V Rousset E Thiéry R A multicentre MLVA and MST typing-ring trial for C. burnetii genotyping: An approach to standardisation of methods5th MedVetNet Annual Scientific Conference 2009 Madrid (Espagne) 03-06 June 2009

[B21] Chmielewski T Sidi-Boumedine K Duquesne V Podsiadly E Thiery R Tylewska-Wierzbanowska S Molecular epidemiology of Q fever in Poland Pol J Microbiol 2009 58 1 9 13 19469280

[B22] Whelan J Schimmer B De Bruin A Van Beest Holle M Van Der Hoek W Ter Schegget R Visits on ’lamb-viewing days' at a sheep farm open to the public was a risk factor for Q fever in 2009 Epidemiol Infect 2012 140 5 858 864 10.1017/S0950268811001427 21835066

[B23] Muskens J van Engelen E van Maanen C Bartels C Lam TJ Prevalence of Coxiella burnetii infection in Dutch dairy herds based on testing bulk tank milk and individual samples by PCR and ELISA Vet Rec 2011 168 3 79 10.1136/vr.c6106 21257587

[B24] Klaassen CH Nabuurs-Franssen MH Tilburg JJ Hamans MA Horrevorts AM Multigenotype Q fever outbreak, the Netherlands Emerg Infect Dis 2009 15 4 613 614 10.3201/eid1504.081612 19331749 PMC2671457

[B25] Benson G Tandem repeats finder: a program to analyze DNA sequences Nucleic Acids Res 1999 27 2 573 580 10.1093/nar/27.2.573 9862982 PMC148217

